# Micro-computed tomography for the quantification of blocked fibers in hemodialyzers

**DOI:** 10.1038/s41598-018-20898-w

**Published:** 2018-02-08

**Authors:** Floris Vanommeslaeghe, Wim Van Biesen, Manuel Dierick, Matthieu Boone, Annemieke Dhondt, Sunny Eloot

**Affiliations:** 1 Nephrology Department, Ghent University Hospital, Ghent, Belgium; 2 UGCT, Department of Subatomic and Radiation Physics, Ghent University, Ghent, Belgium

## Abstract

A novel technique based on micro-CT scanning is developed to quantify coagulation in fibers of hemodialyzers. This objectivation is needed to allow accurate assessment of thrombogenicity of dialyzers used during hemodialysis, for example when comparing different strategies to avoid coagulation and/or fiber blocking. The protocol allowed imaging at a resolution of 25 µm, making it possible to count the open, non-coagulated fibers in a non-invasive way. In 3 fresh, non-used FX600 hemodialyzers, patent fiber counts were extremely consistent (10748 ± 2). To illustrate the potential of this technique, different dialysis parameters currently used as surrogates for fiber blocking were evaluated during 20 hemodialysis sessions. After dialysis, the FX600 dialyzers were visually scored for clotting, dried and subsequently weighed and scanned. The number of patent fibers (10003 [8763,10330], range 534–10692) did not correlate with any of the recorded surrogate parameters. Micro-CT scanning is a feasible, objective, non-invasive, accurate and reproducible tool for quantification of the degree of fiber blocking in a hemodialyzer after use, making it a potential gold standard for use in studies on fiber blocking during renal replacement therapies.

## Introduction

Over 2 million people worldwide require renal replacement therapy to sustain life. The vast majority of them are treated with hemodialysis, a technique in which patient’s blood is purified by circulating it through an extracorporeal circuit containing a hemodialyzer, consisting of around 10000 semi-permeable capillary fibers. The patient’s blood flows in the fiber lumen, while an electrolyte water solution (dialysate) flows counter-currently around the fibers to optimize the diffusive concentration gradient necessary to drive solute transport. Disordered blood flow and contact of the blood with the bio-incompatible material of the fibers activate the coagulation cascade leading to clotting and subsequent blocking of the capillary fibers^1^. This results in a reduction of the efficiency of the dialysis procedure, as less fibers remain available for solute exchange. When more pronounced, coagulation can even cause loss of the extracorporeal blood volume by complete clotting of the extracorporeal circuit, so blood can no longer be returned to the patient. To avoid clotting, appropriate anti-coagulant measures must be applied. Hemodialyzers can be constructed to minimally activate coagulation, either by their design or by using more biocompatible materials. In addition, substances with anticoagulant properties can be administered during the dialysis session. However, overdosing of these anticoagulants can cause bleeding and finding the right balance between under- and over-anticoagulation is challenging.

So far, assessment of the degree of fiber blocking during hemodialysis is measured with different surrogate parameters, but none of them is objective or validated against a gold standard. Some of these are available online during the treatment, such as the dialysis machine parameters venous pressure^2,3^, transmembrane pressure^4^ and online solute clearance^5^. Others are assessed offline, such as visual^6–10^ or software assisted^7^ dialyzer redness scoring. Also laboratory parameters representing coagulation activity in general^6–8,11–13^, measured solute clearance^6,14^, evaluation of rinse-back^3^ or fiber bundle volumes^15–17^ and electron microscopy^9^ have been used. None of these parameters is robust, objective or accurate, which limits the comparative study of different strategies to reduce coagulation and fiber blocking. There is thus a need for an objective, operator-independent and reproducible gold standard to determine the extent of fiber blocking during dialysis treatment^18^. The number of blocked capillary fibers most objectively represents the clinical impact of coagulation.

The present study examines the feasibility and accuracy of micro-computed tomography (micro-CT) to quantify the number of blocked fibers. As illustration, the number of blocked fibers as measured by micro-CT is compared to other surrogate parameters used to assess fiber blocking.

## Results

Micro-CT scanning allowed to acquire high quality images of cross-sections of all scanned dialyzers. Individual capillary fibers could easily be distinguished and their total number quantified. Fiber counting in corresponding cross-sections of the 3 non-used dialyzers resulted in almost exactly the same number of fibers (e.g. 10748 ± 2 in the reference slice), as is expected taking into account the high consistency of the manufactural process of bundling fibers in individual dialyzer capsules.

**Figure 1:**
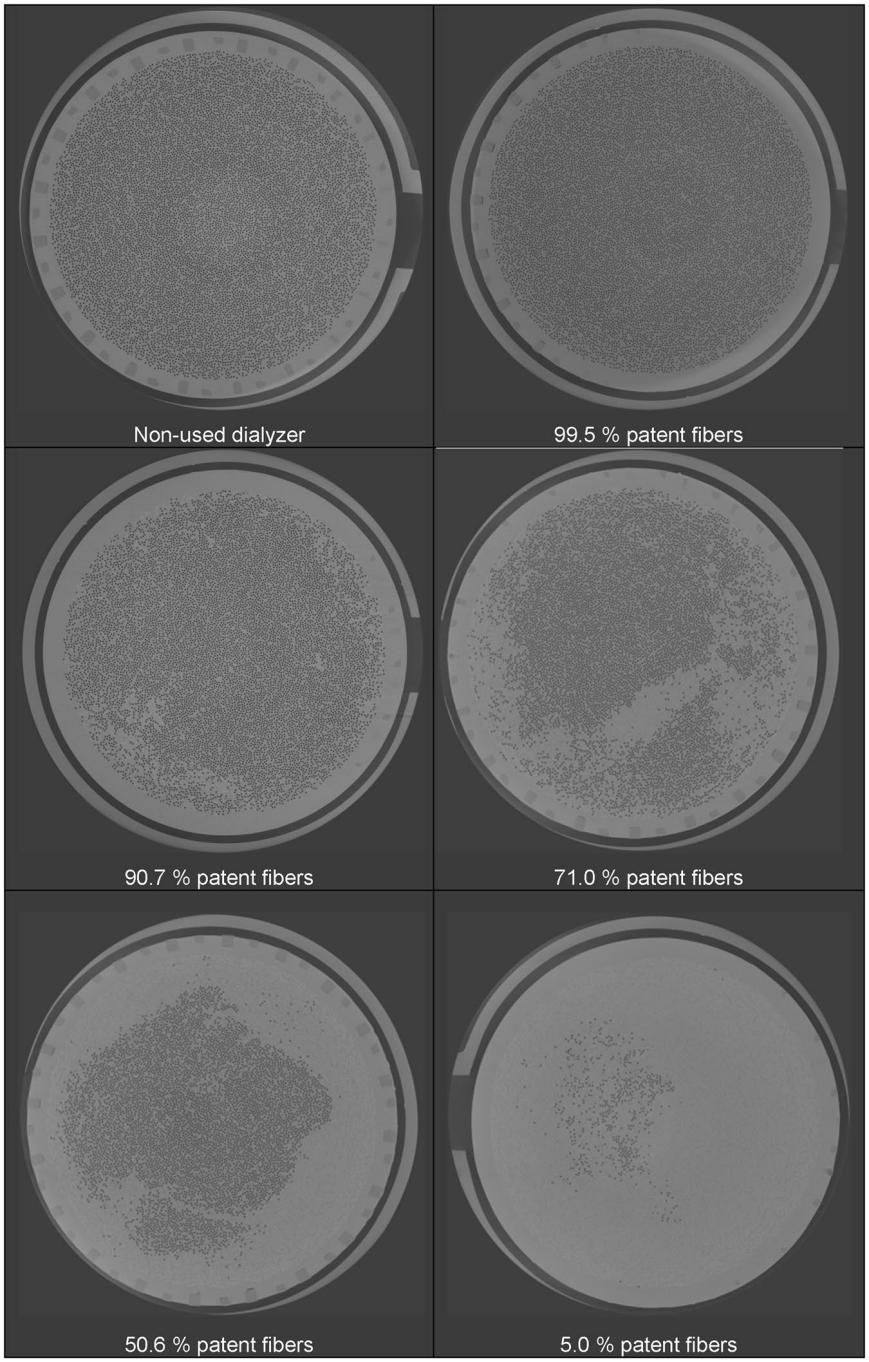
Representative CT images of dialyzer cross-sections (reference slice) showing different levels of coagulation.

Figure 1 shows images representing different levels of coagulation in different used dialyzers. Complete circuit blocking because of dialyzer clotting did not occur during the sessions of the current experiments, but the 20 dialyzers under study represented a broad spectrum of coagulation activation, as reflected by the wide range (534 to 10692) of fibers remaining open after the dialysis session.

Table 1 shows the number of counted open fibers in the non-used and used dialyzers and this in different cross-sections and for different thresholds of open fiber surface areas. Good similarity in counts of open fibers remained when images were assessed using different thresholds to define an ‘open fiber’ (i.e. difference ≤1.7%), showing the robustness and validity of the technique. Differences in the number of open fibers as counted in different cross-sections in the potting were within the clinical relevance (≤6.1%), endorsing the reliability of the technique.

**Table 1:** Number of open fibers as counted in the different scanned dialyzers (percentage of difference in open fibers *versus* those in the reference slice, and related to the total number of fibers).

Dialyzer	Reference slice, threshold 70%	2.5 mm from reference slice, threshold 70%	Reference slice, threshold 60%	Reference slice, threshold 80%
NON-USED DIALYZERS				
Blanco 1	10746	10748 (0)	10749 (0)	10743 (0)
Blanco 2	10750	10751 (0)	10751 (0)	10749 (0)
Blanco 3	10749	10749 (0)	10751 (0)	10745 (0)
Mean ± SD	10748 ± 2	10749 ± 2	10751 ± 1	10745 ± 3
USED DIALYZERS				
1	5443	5497 (0.5)	5478 (0.3)	5391 (0.5)
2	7632	8290 (6.1)	7645 (0.1)	7615 (0.2)
3	10328	10477 (1.4)	10329 (0)	10321 (0.1)
4	10246	10305 (0.5)	10256 (0.1)	10215 (0.3)
5	10335	10368 (0.3)	10336 (0)	10323 (0.1)
6	10228	10391 (1.5)	10232 (0)	10219 (0.1)
7	8327	8335 (0.1)	8335 (0.1)	8258 (0.6)
8	534	558 (0.2)	544 (0.1)	352 (1.7)
9	10041	10134 (0.9)	10043 (0)	10030 (0.1)
10	9451	9118 (3.1)	9461 (0.1)	9442 (0.1)
11	10234	10293 (0.5)	10283 (0.5)	10106 (1.2)
12	6066	6323 (2.5)	6093 (0.4)	5998 (0.5)
13	10692	10688 (0)	10695 (0)	10682 (0.1)
14	10587	10656 (0.6)	10588 (0)	10578 (0.1)
15	8908	9330 (3.9)	8968 (0.6)	8800 (1.0)
16	10351	10419 (0.6)	10364 (0.1)	10299 (0.5)
17	8994	8406 (5.5)	9019 (0.2)	8966 (0.3)
18	10465	10535 (0.7)	10467 (0)	10451 (0.1)
19	9965	9903 (0.6)	9974 (0.1)	9936 (0.3)
20	9752	9791 (0.4)	9764 (0.1)	9707 (0.4)
Median [IQR]	10003 [8763;10330]	10019 [8388;10398]	10009 [8810;10331]	9983 [8665;10305]

**Figure 2:**
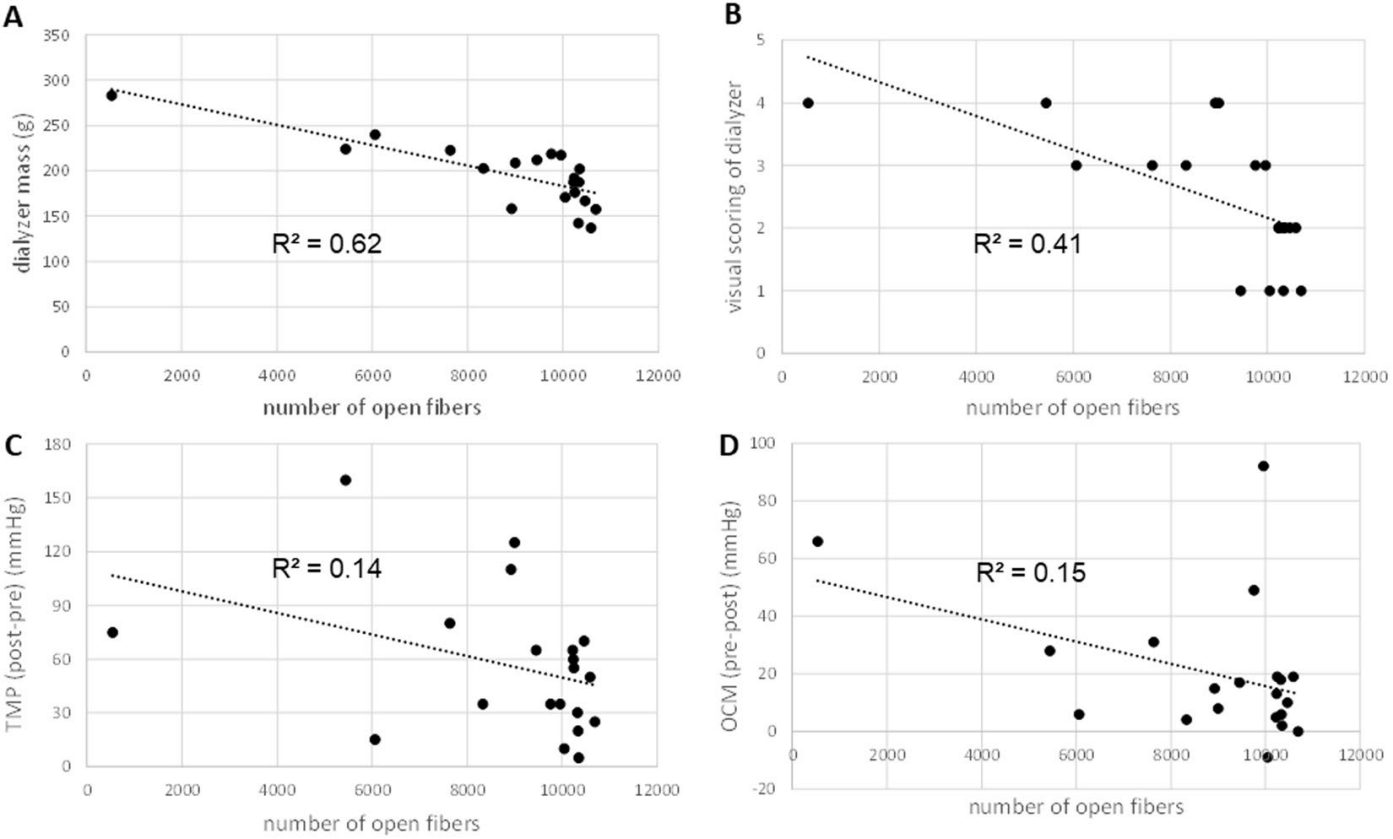
Associations between the number of fibers and a selection of different registered parameters: dialyzer dry mass (panel A), visual scoring of dialyzer (panel B), post minus pre dialysis TMP (panel C), and pre minus post dialysis OCM (panel D).

Associations were found between the number of open fibers and the post-dialysis dry mass of the dialyzers (R² = 0.62), and the visual clotting scores of the dialyzers (R² = 0.41) and venous chambers (R² = 0.34). However, none of the online available parameters as indicated on the dialysis machine (i.e. pressures, blood volume monitoring and online clearance monitoring) as well as the post dialysis registered rinse-back volume were associated with the number of open fibers (Table 2 and Fig. 2).

**Table 2:** Absolute values as well as regression coefficients (R²) of the associations of the number of open fibers with the post dialysis measurements and scorings, and with the interdialytic changes in registered machine parameters in the 20 dialysis sessions.

Dialyzer	#fibers ref slice 70% threshold	dialyzer dry mass (g)	visual scoring dialyzer	visual scoring venous chamber	Rinse back volume (mL)	arterial pressure pre-post (mmHg)	venous pressure pre-post (mmHg)	TMP post-pre (mmHg)	BVM pre-post (%)	OCM pre-post (mL/min)
1	5443	224.0	4	3	220	25	−5	160	7	28
2	7632	222.5	3	2	280	0	−45	80	9	31
3	10328	142.0	1	1	280	10	20	30	4	18
4	10246	176.0	2	1	270	10	−105	55	11	19
5	10335	187.5	2	2	270	30	15	20	6	6
6	10228	187.5	2	2	270	−25	0	65	6	5
7	8327	202.5	3	2	260	25	10	35	5	4
8	534	283.0	4	3	360	60	−35	75	20	66
9	10041	171.0	1	1	260	55	−20	10	12	−9
10	9451	212.0	1	1	270	15	40	65	10	17
11	10234	192.0	2	2	260	65	30	60	9	13
12	6066	240.0	3	3	270	−5	75	15	3	6
13	10692	157.5	1	2	270	15	5	25	7	0
14	10587	137.0	2	2	290	10	0	50	5	19
15	8921	158.0	4	2	260	−10	5	110	5	15
16	10351	202.0	2	1	310	−35	5	5	−2	2
17	8998	209.0	4	1	260	−10	15	125	6	8
18	10465	167.0	2	2	270	20	20	70	13	10
19	9965	217.0	3	3	250	65	45	35	8	92
20	9752	218.5	3	1	260	35	20	35	13	49
R² (with #fibers):		**0.62** ^*^	**0.41** ^*^	**0.34** ^*^	**0.20**	**0.04**	**0.01**	**0.14**	**0.18**	**0.15**

^*^p < 0.05; ^#^number; TMP: transmembrane pressure; BVM: blood volume monitoring; OCM: online clearance monitoring.

## Discussion

We demonstrate the feasibility to use a self-developed micro-CT technique to assess objectively and in an operator-independent manner the blocking of individual fibers in hemodialyzers with great accuracy and repeatability. Using this technique, we demonstrate that currently used parameters, based on data derived from the dialysis monitor, visual scoring or post-dialysis mass of the dialyzer are not representative to assess the degree of fiber blocking.

The circulation of blood through extracorporeal devices such as hemodialyzers activates the coagulation cascade, which might result in clotting and obstruction of the circuit. In hemodialysis, this progressive blocking of fibers within the dialyzer results in a progressive decrease of dialysis performance, and potential loss of blood when the circuit becomes completely obstructed. The degree of activation of the coagulation cascade depends on the biocompatibility of the dialyzer and the coagulability of the blood. Biocompatibility is in itself a combination of design, for example geometry to avoid turbulence, blood stagnation and shear stress, and the type of materials used^19,20^. Coagulability depends upon patient factors, for example presence of inflammation or coagulation disorders, and can be influenced by applying an anticoagulant strategy during the dialysis session. This should however be done with caution, as too much anticoagulation might induce bleeding and death of the patient.

Different designs of filters and anticoagulant strategies have been developed, but comparing the balance of efficacy and safety between different designs and strategies is hampered by the lack of a well-established and objective tool to evaluate the resulting degree of filter blocking in the hemodialyzer. There is thus need for a gold standard to assess and compare thrombogenicity of different dialyzers and/or anticoagulation strategies. Ideally, such technique quantifies the patency of the individual fibers in the complete dialyzer, to assess the impact of coagulation on the dialyzer performance. The technique should be repeatable, accurate and operator-independent (objective). The here presented micro-CT scanning technique fulfills these criteria. It allows consistent and reliable counting of open *versus* blocked fibers in an accurate and repeatable way, as demonstrated by the agreement of measurements at different cross-sections of the dialyzer. In addition, this high accuracy is independent of the threshold chosen to define patency of fibers.

The present technique offers multiple advantages over the currently available methods to assess the degree of fiber blocking. So far, visual assessment has been most frequently promoted to assess coagulation of dialyzers. In this technique, a visual assessment of the discoloration of the fibers from white to red by residual (clotted and blocking) blood is done to estimate the degree of fiber blocking within the dialyzer. Despite existence of tools to enhance standardization, such as calibrated reference pictures, this simple method remains operator-dependent. This operator-dependency can theoretically be decreased by using computer analysis of pictures of the dialyzer rather than visual assessment by the human eye. However, still then the technique allows only assessment of fibers at the outside of the dialyzer, as more internal fibers cannot be visualized. As coagulation is not homogenous, this can result in big discrepancies between estimated and true degree of coagulation. This is more of importance as coagulation can occur at specific areas exactly because of the design of the dialyzer, for example at places of high turbulence, or stagnation of flow. The lack of capacity to assess the complete cross-section by visual assessment can thus lead to misinterpretation of the advantage/disadvantage of certain designs or strategies. Micro-CT evaluation is performed on complete sections of the dialyzer, allowing assessment of all fibers, irrespective of their location. Last, visual scoring of clotting can be hampered by non-transparent fiber and housing design, and can thus not be used in all types of dialyzers while the presented micro-CT technique is independent of the type of housing. In our study, there was a relatively good association between the visual score of the hemodialyzer and the number of blocked fibers (R² = 0.36) (Table 2 and Fig. 2 panel B). However, the point prevalence power of the visual score to predict the number of open fibers in individual dialyzers was disappointing. For example, a visual score of 3 corresponded with 6000 in one and 10000 open fibers in another dialyzer, whereas 10000 open fibers resulted in a visual score of 1 in one and 3 in another dialyzer (Fig. 2 panel B), making that visual scoring is completely not reliable at the level of the individual patient.

Dialyzer mass after post dialysis rinsing could be used as a proxy of blocked fibers. This method is based on the postulate that it is impossible to remove debris and blood from blocked fibers, so that more mass corresponds with more blocked fibers. In our experiments, post dialysis dialyzer mass corresponded best of all parameters with the number of open fibers as assessed by micro-CT, with an R² of 0.62 (Table 2 and Fig. 2 panel A). However, also here, the point prevalence predictive power was disappointing and insufficient to accept post dialysis dialyzer mass as an acceptable surrogate for degree of fiber blocking.

Laboratory markers for coagulation activation have been used to assess degree of coagulation during a hemodialysis session. However, there is a lack of standardization of how these tests should be performed and how results should be interpreted. More important, the results of these tests are representing coagulation in the complete system, including the patient. As such, it is unclear from these tests where exactly the coagulation cascade has been activated, either in the patient, for example due to inflammation, or in the dialyzer. As a consequence, these tests are less informative about the impact of dialyzer design or anticoagulation strategy on the blocking of fibers.

In many centers, parameters that are routinely available from the dialysis machine, such as venous pressure and estimated transmembrane pressure are being used to assess the degree of fiber blocking. From the regression analyses, we found that none of these monitor derived parameters accurately reflects degree of coagulation. Pre-dialyzer pressure and measured transmembrane pressure potentially would perform better, but none of these parameters is provided on the presently commercially available dialysis machines.

Also the volume needed to achieve clean rinse-back, as determined by a sensor on the venous blood line or chamber, seems not to be able to predict the number of blocked fibers. This corresponds with the observation that visual inspection of blood lines and chambers does not necessarily reflect coagulation within the dialyzer.

This study also showed for the first time that online clearance monitoring (OCM), claimed to be an indicator of dialysis performance, does not correspond with the degree of fiber blocking during dialysis. For example, in a dialyzer ending up with only 534 patent fibers out of more than 10000 at start, (as shown in Fig. 1, lower right image), OCM values were only decreased by 28% after the 4 hour dialysis session. Although dialyzer clotting is an important issue in clinical practice, dialysis machines do thus not provide any parameter to accurately predict progressive coagulation in the extracorporeal circuit.

The micro-CT technique does not allow mechanistic assessment of underlying causes of fiber blocking at the molecular level. This would require much higher resolutions than what is achieved in the current set-up. Such higher resolution would be at the expense of the fact that the current set-up of the micro-CT scan allows to visualize the complete cross-section of the dialyzer, as it would necessitate analysis of processes at the level of membrane pores. As the main goal of the current micro-CT set-up was to evaluate the impact of different dialyzer designs and/or anticoagulation strategies on the clinical performance of the dialyzer, the current resolution allowing to determine blocking at the level of individual fibers is sufficient. However, the current set-up could also be used to indirectly explore underlying pathophysiology of coagulation, by comparing different strategies, for example with or without addition of antibodies^21^ blocking specific coagulation pathways, and assess their impact on filter blocking.

In conclusion, there is a need for a gold standard to assess blocking of hemodialyzers at the level of individual fibers if we want to evaluate progress in the development of new strategies to reduce coagulation during hemodialysis. Advantage of strategies or designs claiming to improve anticoagulation over existing practice can be demonstrated or refuted by using this gold standard. The micro-CT approach also offers new perspectives for research on coagulation regimens in renal replacement therapies. Much needed online surrogate markers of coagulation can be validated against this method.

## Patients and Methods

This single center, open-label, non-controlled prospective trial included twenty adult patients (11 male) undergoing 245 ± 20 min maintenance hemodialysis (pressure controlled on-line postdilution hemodiafiltration, replacement fluid 20 ± 4 L). The median age was 75 [69–79]. Median weight was 65 [58–72] kg. Diabetic and hypertensive nephropathy were the most frequent underlying diseases. Sixty percent of the patients received antiplatelet therapy. Table 3 lists the patient’s clinical characteristics and baseline laboratory findings.

**Table 3:** Patient characteristics and baseline laboratory data.

	Median [Q1; Q3] and (min; max) or n (%)
Cause of ESRD no. (%)	
Diabetic/hypertensive nephropathy	7 (35%)
Post-Renal Causes	3 (15%)
Glomerulonephritis	4 (20%)
Others	6 (30%)
Co-existing illnesses no. (%)	
Smoking	2 (10%)
History of cancer	7 (35%)
Diabetes	6 (30%)
Thromboembolic Venous Disease	5 (25%)
Antithrombic therapy—no. (%)	
Aspirine (monotherapy)	11 (55%)
Clopidogrel (monotherapy)	0
Aspirine and Clopidogrel	1 (5%)
Vitamin K antagonist	3 (15%)
Dialysis LMWH anticoagulation	
No anticoagulation	1 (5%)
Enoxaparin 20 mg	1 (5%)
Enoxaparin 40 mg	4 (20%)
Enoxaparin 60 mg	4 (20%)
Tinzaparin 2500 U	2 (10%)
Tinzaparin 3500 U	5 (25%)
Tinzaparin 4500 U	3 (15%)
Clotting Parameters (mean + −SD)	
Hemoglobin, g/dl	9.8 [8.7–11.8] (7.9–13.5)
Hematocrit, %	31.0 [27.7–36.0] (22.8–41.6)
Platelets, x 10E3/µL	179 [115–255] (43–431)
Prothrombin time (PT)	86 [69–102] (21–115)
International Normalized Ratio (INR)	1.1 [1.0–1.3] (0.9–3.6)
Access	
Arteriovenous fistula	7 (35%)
Permanent dialysis catheter	13 (65%)

The protocol was approved by the ethics committee of the Ghent University Hospital, and written informed consent was obtained from all included patients or their legal representatives (EC 2016/0908–B670201629247).

### Dialysis and anticoagulation

All dialysis treatments were performed in accordance with the relevant guidelines and regulations. Patients received their standard Low-Molecular-Weight Heparin (Enoxaparin, Sanofi Belgium or Tinzaparin, Leo Pharma, Belgium) anticoagulation at the beginning of the dialysis session. All sessions were performed with single-use high flux FX600 Cordiax® polysulfone dialyzers with a surface area of 1.6 m² on 5008 dialysis machines (Both Fresenius Medical Care, Bad Homburg, Germany). Blood flow was maintained between 200 and 350 ml/min (297 ± 43), while ultrafiltration was set according to the patient’s interdialytic weight gain and clinical status (509 ± 194 ml/h). In all dialysis sessions, double-needle vascular access was achieved through a native arteriovenous fistula or a well-functioning double lumen tunneled central venous catheter.

### Online dialysis machine parameters

Different dialysis parameters as reported by the dialysis machine were registered every 30 min: i.e. venous and arterial pressure, transmembrane pressure (TMP), blood volume monitoring (BVM) and online clearance monitoring (OCM). For further regression analyses, absolute changes, calculated as post *versus* predialysis values, were used. Rinse-back volume, representing the volume of dialysate needed to restore all extracorporeal blood back to the patient at the end of the session was registered from the dialysis monitor.

### Dialyzer and venous chamber scoring

Hemodialyzers and blood lines were removed immediately once the post-dialysis standard rinsing procedure had been completed. Subsequently, a member of the nursing staff and a clinician independently scored the extent of clotting in the dialyzer and the venous chamber in a semi-quantitative way using a reference scoring system^10^. Dialyzers were graded having no signs of clotting (1), few dark red fibers (2), less than 50% dark red fibers (3) or overall redness with >50% dark red fibers (4). Thrombus formation in the air bubble catcher (venous chamber) was quoted using a four graded scale: no detectable clotting (1), minimal clot formation (2), clots up to 5 cm but dialysis still possible (3), and complete occlusion of the air bubble catcher (4) (Table 4).

**Table 4:** Visual clotting scores.

Clotting score dialyzer		Number of dialyzers (%)
1	Clean filter	4 (20)
2	Few dark red fibers	7 (35)
3	<50% dark red fibers	5 (25)
4	>50% dark red fibers	4 (20)
Venous chamber scoring		
1	no detectable clotting	7 (35)
2	minimal clot formation	9 (45)
3	clots up to 5 cm but dialysis still possible	4 (20)
4	complete occlusion of the air trap	0 (0)

Next, in an *in vitro* setting, continuous subtle positive pressure ventilation was applied to the dialyzer both through the blood inlet as through the dialysate outlet for 24 hours, to dry the dialyzer membrane. Finally, dialyzer dry mass was measured using a precise scale (CT 6000, Ohaus®, USA - calibration by Eldon Enterprises France).

### Micro-CT scanning

**Figure 3:**
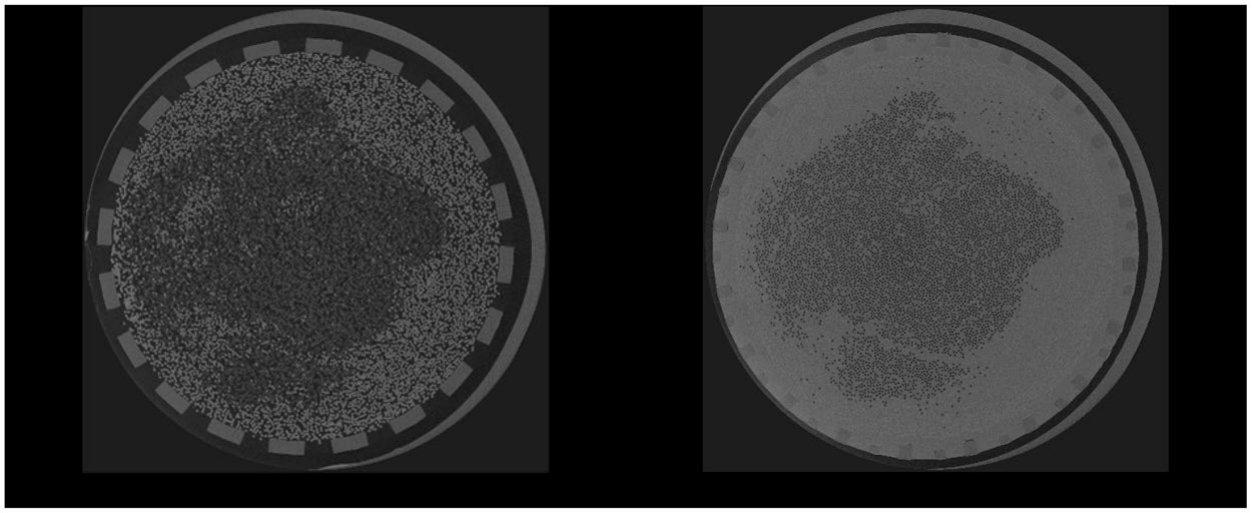
CT images of cross-sections in the same used dialyzer halfway the dialyzer (left panel) and at the central cross-section in the outlet potting (right panel). Central fibers are open and peripheral fibers are blocked. Sections in the potting offer optimal visualization of air inflated open fibers, appearing as black dots.

Dialyzer fiber clotting was visualized using a 3D CT scanning technique on micrometer resolution. HECTOR is a High Energy CT scanner Optimized for Research^22^, built by the Ghent University Centre for X-ray Tomography (UGCT) in collaboration with the UGCT spin-off company XRE (Gent, Belgium). In front of the X-ray source, the dialyzer was mounted vertically on a precision rotation stage, and radiographies were recorded over 360° with an angular interval of 0.15°. Scan conditions were optimized to maximize the signal-to-noise ratio based on the sample size and structure, and the scanner properties. The tube voltage was set at 80 kV, at a power of 20 Watts, the maximal power that allowed imaging at a resolution of 25 µm. A total of 2401 projections were recorded with 500 ms exposure each, resulting in a total exposure time of 20 minutes. Acquired images at 0 (projection 1) and 360° (projection 2401) were compared to exclude movement of the hemodialyzer during the scanning process. Reconstruction of the raw projection data is performed with the Octopus Reconstruction software package, licensed by XRE^23^. This resulted in two types of images as presented in Fig. 3: fibers are visible as rings for cross-sections in the free fiber bundle (left panel), and as black dots for cross-sections in the dialyzer potting (right panel), a polyurethane sealing near the blood inlet and outlet, fixing the ends of the capillary fibers.

### Fiber counting and coagulation quantification

Fibers were counted in the central cross-section of the dialyzer outlet potting (reference slice) since this region offers optimal visualization of air inflated open fibers, appearing as black dots (Fig. 3–right panel). These dots can easily be counted in a computer-based way, in contrast to the use of cross-sectional images taken out of the potting showing fibers as white cylinders (Fig. 3–left panel).

**Figure 4:**
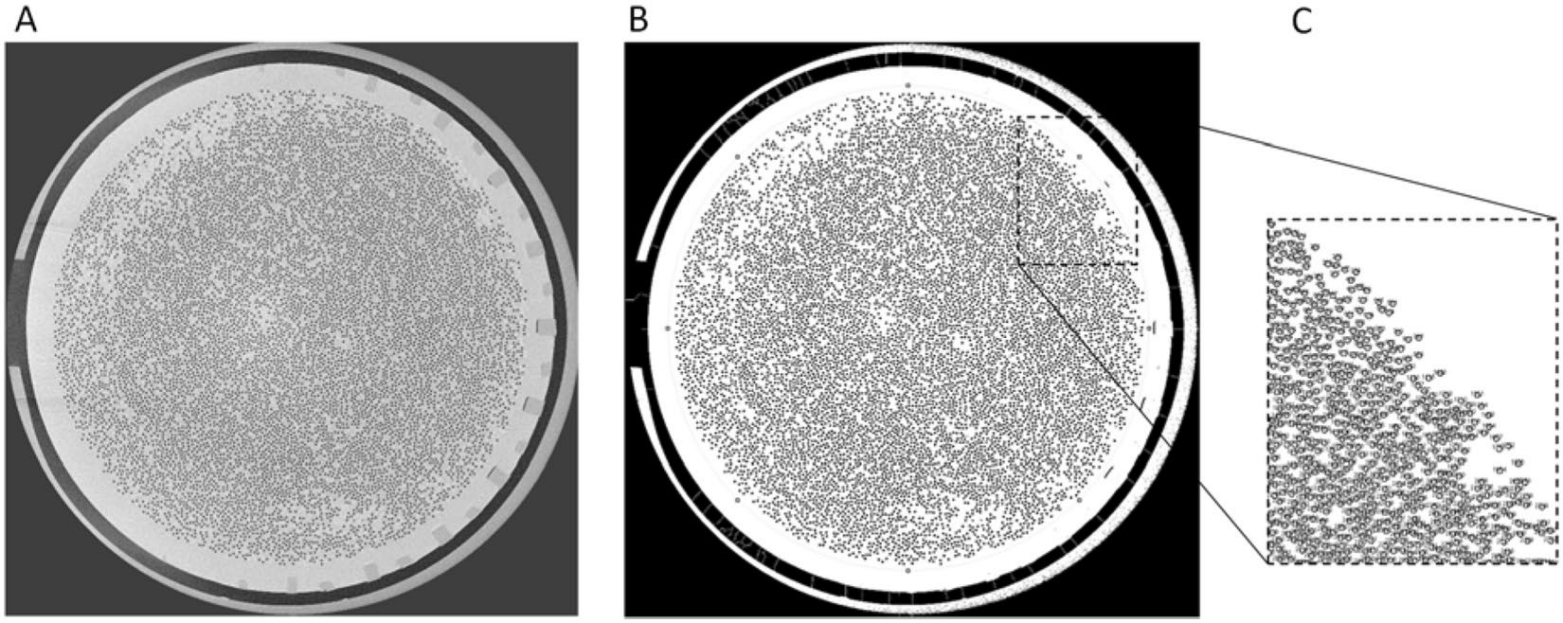
CT image of cross-section (panel A). Optimization of image brightness and selection of the region of interest (ROI) bordering the entire fiber bundle (panel B). Zoomed inset of counted fibers (panel C).

The non-coagulated fibers (i.e. black dots) were counted using the Fiji image processing toolkit of ImageJ analysis software^24,25^ (ImageJ 1.51 H, NIH, Bethesda, USA), an open-source platform for biological-image analysis^26^ (Fig. 4). To ameliorate selection and count of the patent fibers, the brightness of each individual image was optimized using image thresholding^27^ (Fig. 4–panel B). The region of interest (ROI) was selected bordering the entire fiber bundle to avoid count of any disturbances outside the fiber bundle (Fig. 4–panel B). Watershed segmentation was employed to distinguish sticky fibers^28^. Image particles with a circularity of 0.5–1 were selected (Fig. 4–panel C).

Based on the reference images of the non-used dialyzers, only fibers with a surface area of >70% (threshold) of the cross section of a non-used fiber were considered to be open. This correlates with a surface area of 0.0204 mm². Validity and reliability of the method were checked, the former by comparing the impact of different arbitrary thresholds (60 and 80% open fiber) to define open fibers, the latter by performing counts in a cross-section 100 slices (i.e. 2.5 mm) proximal of the reference slice.

### Statistical analysis

Statistical analyses were performed using SPSS (version 24, SPSS Inc, Chicago, USA). Continuous variables were summarized as mean ± SD if normally distributed (normality was checked with Shapiro-Wilk test); otherwise median value with interquartile range [IQR] were reported. Categorical variables were expressed as frequencies and percentages. Regression analyses were performed (R²) with the number of open fibers as independent variable and the dialysis parameters as dependent variables.
